# Supplementary material for: Functional Analyses of Four CYP1A1 Missense Mutations Present in Patients with Atypical Femoral Fractures
Source: Int J Mol Sci. 2021 Jul 9;22(14):7395. doi: 10.3390/ijms22147395 (PMC8303772; doi:10.3390/ijms22147395)
Supplement: Supplementary file 1 [file ijms-22-07395-s001.zip › Table S1.pdf]

Table S1. Primers used in this study

Primers for site-directed mutagenesis		
p.Arg98Trp	Fw	CATCGCCCTGCC**A**CACCAGGCCTG
	Rv	CAGGCCCTGGTG**T**GGCAGGGCGATG
p.Arg136His	Fw	CTGGCCAGG**T**GCCGGCGGGC
	Rv	GCCCGCCGGC**A**CCTGGCCCAG
p.Ser216Cys	Fw	GGTTGACTAGGC**A**AAGCAGTTCTTGGTGGTTGTGG
	Rv	CCACAACCACCAAGAACTGCTT**T**GCCTAGTCAACC
p.Val409Ile	Fw	AGGGGCGTTGTGTCTTT**A**TAAACCAGTGGCAGATC
	Rv	GATCTGCCACTGGTTTA**T**AAAGACACAACGCCCCT
Primers for qPCR		
CYP1A1	Taqman assay	Hs01054796_g1
GAPDH	Fw	CCCCGGTTTCTATAAATTGAGC
	Rv	CTTCCCCATGGTGTCTGAG

The mutated nucleotides are indicated in bold
