# Supplementary figures and images for: Functional Analyses of Four CYP1A1 Missense Mutations Present in Patients with Atypical Femoral Fractures
Source: Int J Mol Sci. 2021 Jul 9;22(14):7395. doi: 10.3390/ijms22147395 (PMC8303772; doi:10.3390/ijms22147395)

# CYP1A1

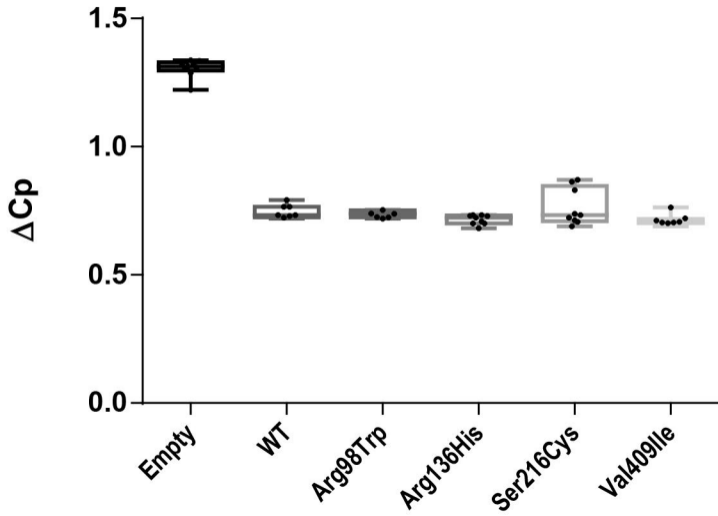

Supplement: Supplementary file 1 [file ijms-22-07395-s001.zip › FigS1.pdf]
